# Association of Bovine Leukemia Virus-Induced Lymphoma with BoLA-DRB3 Polymorphisms at DNA, Amino Acid, and Binding Pocket Property Levels

**DOI:** 10.3390/pathogens10040437

**Published:** 2021-04-06

**Authors:** Chieh-Wen Lo, Shin-nosuke Takeshima, Kosuke Okada, Etsuko Saitou, Tatsuo Fujita, Yasunobu Matsumoto, Satoshi Wada, Hidetoshi Inoko, Yoko Aida

**Affiliations:** 1 Laboratory of Global Animal Resource Science, Graduate School of Agricultural and Life Sciences, The University of Tokyo, 1-1-1 Yayoi, Bunkyo-ku, Tokyo 113-8657, Japan; rogerwen80@gmail.com (C.-W.L.); matsumoto-yasu@mvi.biglobe.ne.jp (Y.M.); 2 Laboratory of Global Infectious Diseases Control Science, Graduate School of Agricultural and Life Sciences, The University of Tokyo, 1-1-1 Yayoi, Bunkyo-ku, Tokyo 113-8657, Japan; 3 Viral Infectious Diseases Unit, RIKEN, 2-1 Hirosawa, Wako, Saitama 351-0198, Japan; takesima@jumonji-u.ac.jp; 4 Department of Food and Nutrition, Jumonji University, Niiza, Saitama 352-8510, Japan; 5 Iwate University, 7-360 Mukai-shinden Ukai, Takizawa, Iwate 020-0667, Japan; kosuke@iwate-u.ac.jp; 6 Hyogo Prefectural Awaji Meat Inspection Center, 49-18 Shitoorinagata, Minamiawaji, Hyogo 656-0152, Japan; Etsuko_Saitou@pref.hyogo.lg.jp; 7 Livestock Research Institute of Oita Prefectural Agriculture, Forestry and Fisheries, Research Center, Kuju, Taketa, Oita 878-0201, Japan; pu1508708@pref.oita.jp; 8 Photonics Control Technology Team, RIKEN Center for Advanced Photonics, Wako 351-0198, Japan; swada@riken.jp; 9 Genome Analysis Division, GenoDive Pharma Inc., 4-14-1 Nakamachi, Atsugi-shi, Kanagawa 243-0018, Japan; hinoko@is.icc.u-tokai.ac.jp; 10 Benno Laboratory, Baton Zone Program, RIKEN Cluster for Science, Technology and Innovation Hub, 2-1 Hirosawa, Wako, Saitama 351-0198, Japan

**Keywords:** bovine leukemia virus, lymphoma, *BoLA-DRB3*, polymorphisms, antigen recognition sites, amino acid motif, peptide-binding pockets, electrostatic charge, association study

## Abstract

Bovine leukemia virus (BLV) causes enzootic bovine leucosis, a malignant B-cell lymphoma in cattle. The DNA sequence polymorphisms of bovine leukocyte antigen (BoLA)*-DRB3* have exhibited a correlation with BLV-induced lymphoma in Holstein cows. However, the association may vary between different cattle breeds. Furthermore, little is known about the relationship between BLV-induced lymphoma and DRB3 at the amino acid and structural diversity levels. Here, we comprehensively analyzed the correlation between BLV-induced lymphoma and DRB3 at DNA, amino acid, and binding pocket property levels, using 106 BLV-infected asymptomatic and 227 BLV-induced lymphoma Japanese black cattle samples. DRB3*011:01 was identified as a resistance allele, whereas DRB3*005:02 and DRB3*016:01 were susceptibility alleles. Amino acid association studies showed that positions 9, 11, 13, 26, 30, 47, 57, 70, 71, 74, 78, and 86 were associated with lymphoma susceptibility. Structure and electrostatic charge modeling further indicated that binding pocket 9 of resistance DRB3 was positively charged. In contrast, alleles susceptible to lymphoma were neutrally charged. Altogether, this is the first association study of *BoLA-DRB3* polymorphisms with BLV-induced lymphoma in Japanese black cattle. In addition, our results further contribute to understanding the mechanisms regarding how *BoLA-DRB3* polymorphisms mediate susceptibility to BLV-induced lymphoma.

## 1. Introduction

Enzootic bovine leucosis (EBL) is a lymphoproliferative disease characterized by B-cell lymphoma and is the most common neoplasm disease in cattle [1]. Bovine leukemia virus (BLV) infection is the causative agent of EBL [2]. As EBL is a lethal disease leading to severe financial burden in the cattle industry, it is recognized by the World Organization for Animal Health as a disease of importance for international trade [3]. Recent surveys in most countries worldwide except Europe have reported a continuous increase in BLV infection in cattle [4]. Consequently, it is difficult to develop new strategies to decrease the disease prevalence rate, as the mechanisms of BLV-induced lymphoma are unknown.

BLV belongs to family *Retroviridae* and genus *Deltaretrovirus* and is closely related to the human T-cell leukemia viruses [5]. Like other retroviruses, the DNA copies of BLV RNA genome integrate into the host genome as a provirus and induce lifelong infection. In the majority of cases, approximately 70% of BLV-infected cattle are asymptomatic carriers. Approximately 30% of infected individuals progress to persistent lymphocytosis, characterized by polyclonal expression of the non-neoplastic CD5^+^ B lymphocyte population. Only 1–5% of infections develop B-cell lymphoma after a prolonged latency period [6]. The low mortality rate of BLV infection implies that the virus itself may not be sufficient to induce disease onset and that host genetic polymorphisms in individuals potentially play a key role in disease susceptibility.

The major histocompatibility complex (MHC) is a highly polymorphic gene set responsible for peptide antigen presentation and immune responsiveness and is, therefore, associated with disease susceptibility [7]. Bovine leukocyte antigen (BoLA) is the MHC system in cattle [8]. Specifically, *BoLA-DRB3* is the highly polymorphic *BoLA class II* locus, with 365 alleles registered in the Immuno-Polymorphism Database (IPD)-MHC database (https://www.ebi.ac.uk/ipd/mhc/group/BoLA/) (accessed on 4 April 2021) and is associated with many infectious diseases in cattle [9,10,11]. The associations of *BoLA-DRB3* polymorphisms with BLV pro-viral load (PVL) and related symptoms are well documented [12,13,14]. In fact, *BoLA-DRB3* polymorphisms have been shown to affect BLV PVL regulation in a cattle experimental infection model [15,16], thereby strengthening the importance of *BoLA-DRB3* in BLV transmission and disease progression. Indeed, *BoLA-DRB3* is a promising target for breeding selection to decrease BLV transmission and related pathogenesis [17,18]. However, we recently found that BLV PVL and lymphoma are associated with differential *BoLA-DRB3* polymorphisms in Holstein cows [19], and thus, further research regarding the effect of *BoLA-DRB3* polymorphisms on lymphoma development is warranted. In addition, the associations between *BoLA-DRB3* polymorphisms and BLV-related diseases vary in different breeds and locations of cows. However, information regarding the association between *BoLA-DRB3* polymorphisms and BLV-induced lymphoma is currently only available in Holstein cows, and therefore the relationship requires elucidation in different breeds of cattle, for example, Japanese black cattle. 

A functional BoLA class II DR molecule consists of an α chain and a β chain, that are encoded from a single polymorphic gene, *BoLA-DRA*, and a highly polymorphic gene, *BoLA-DRB3*, respectively. Therefore, the antigen peptide-binding preference of BoLA class II DR molecule is mainly determined by the BoLA-DRβ polymorphisms. The polymorphisms of *BoLA-DRB3* occur in the exon 2 region, encoding the BoLA-DRβ β1 domain, which is the peptide-binding cleft, containing five peptide-binding pockets, 1, 4, 6, 7, and 9, which constitute the structure and govern the binding strength of peptides with BoLA-DRβ [20]. Consequently, the amino acid composition and the chemical/physical property variations of these five binding pockets may largely affect disease susceptibility. It is known that the binding affinity of peptide antigen with MHC class II is one of the factors that determine subsequent T helper cell-mediated immune responses, i.e., T helper type 1 (Th1) and T helper type 2 (Th2) responses [21]. Th1 is known for cell-mediated immunity, secreting interferon-γ (IFN-γ), and efficacy against viruses [22,23]. Furthermore, a recent study indicated that a toll-like receptor 7 agonist could activate bovine Th1 and thus promoted anti-BLV infection in vivo [24]. Th2 is associated with humoral immunity, which is known to be less effective against BLV infection [25]. Indeed, experimental infection in an ovine model demonstrated that amino acids 70 and 71 in the binding pocket 4 of ovine leukocyte antigen (OLA)-DRβ were critical for BLV-induced lymphoma susceptibility or resistance by affecting the efficiency of Th1 activation [26,27]. This result highlights the importance of amino acid and structure analysis of BoLA-DRβ in understanding how BoLA-DRβ polymorphisms affect lymphoma susceptibility. In addition, it is known that differences in the binding pockets property of MHC molecules affect the peptide binding preference. For example, HLA-DQ2 molecules with positively charged binding pockets compared with that with negatively charged binding pockets has better ability at binding with proline-glutamate rich peptide [28]. Therefore, the study of BoLA-DRβ polymorphisms at amino acid and structural levels could contribute to understanding the binding pocket properties of each BoLA-DRβ molecule and potentially provide information for future peptide vaccine development, e.g., the charge of peptide should be taken into account. Although few reports have found an association between the *BoLA-DRB3* allele and BLV-induced lymphoma [19,29], little is known about the relationship between BLV-induced lymphoma and BoLA-DRβ at the amino acid and structure levels. Here, we present the first association study involving BLV-induced lymphoma and *BoLA-DRB3* in Japanese black cattle. In addition, the relationship was comprehensively investigated at the *BoLA-DRB3* DNA sequence, DRβ amino acid, and DRβ binding pocket structural property levels.

## 2. Results

### 2.1. BoLA-DRB3 Genotyping in Asymptomatic and Lymphoma Cattle

Lymphoma cattle blood samples were collected from 227 BLV-infected, disease onset Japanese black cattle. The genotyping was performed in all cattle samples using a PCR-sequence-based typing (SBT) method of *BoLA-DRB3* at exon 2. In total, 21 known alleles were identified in asymptomatic cattle and 24 known alleles were found in lymphoma cattle (Table 1).

### 2.2. Association Study of BoLA-DRB3 with BLV-Induced Lymphoma

An association analysis based on Fisher’s exact test found that DRB3*005:02 (OR = 11.260) and DRB3*016:01 (OR = 2.953) were lymphoma susceptibility alleles, whereas DRB3*011:01 (OR = 0.186) was a lymphoma resistance allele (Table 1). In addition, DRB3*005:03 (OR = 5.106) showed a tendency to lymphoma susceptibility, whereas DRB3*002:01 (OR = 0.419), DRB3*009:02 (OR = 0.130), and DRB3*015:01 (OR = 0.511) showed a tendency to lymphoma resistance, although they did not meet the significance threshold after the stringent adjustment for multiple testing (Bonferroni correction).

It was found that the effect of the resistance allele was dominant over the susceptibility allele in PVL association studies [13]. To address whether this observation was true for lymphoma association, we then performed an association study involving the BoLA-DRB3 genotype with BLV-induced lymphoma (Table 2). Interestingly, only the DRB3*016:01/*016:01 homozygote (OR = 7.020) was found as a susceptibility genotype, but no resistant genotypes were found after Bonferroni correction. This may be due to the high divergent level of BoLA-DRB3 genotypes, but lacked major resistance genotypes. The susceptibility tendency was found in DRB3*005:02/*016:01 (OR = 13.405) and DRB3*005:03/*016:01 (OR = 10.283), whereas resistance tendency was found in DRB3*002:01/*010:01 (OR = 0.180), DRB3*002:01/*011:01 (OR = 0.041), DRB3*011:01/*015:01 (OR = 0.050), and DRB3*011:01/*016:01 (OR = 0.254).

### 2.3. Association Study of BoLA-DRβ with BLV-Induced Lymphoma at the Amino Acid Level

**Figure 1:**
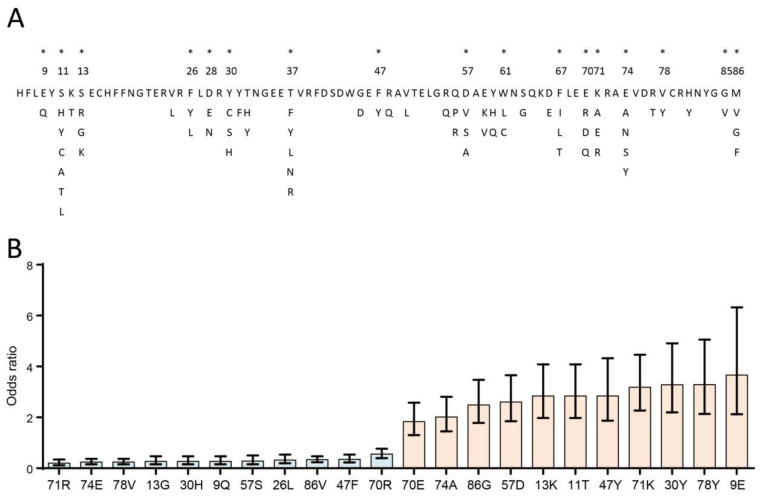
Association of antigen recognition sites of peptide-binding pockets in BoLA-DRβ with lymphoma. (**A**) Amino acid diversities of BoLA-DRβ based on the *BoLA-DRB3* allele, which was identified in this study. The asterisks indicate the antigen recognition sites of BoLA-DRβ, located in the antigen interacting position. (**B**) Odds ratio of BoLA-DRβ binding pocket amino acids and lymphoma. Resistance amino acid: Odds ratio < 1, indicated in blue; Susceptibility amino acids: Odds ratio > 1, indicated in red. The association study was performed using Fisher’s exact test followed by Bonferroni correction. Error bars indicate the 95% confidence intervals. Only Bonferroni-corrected significant amino acids are shown.

The potential mechanisms of allele differential susceptibility in lymphoma are due to variations in antigen recognition sites for their encoding BoLA-DRβ molecules. The sequence of *BoLA-DRB3* at exon 2 allowed us to determine the amino acid variations of 17 antigen recognition sites (positions 9, 11, 13, 26, 28, 30, 37, 47, 57, 61, 67, 70, 71, 74, 78, 85, and 86), which are located within the five peptide-binding pocket regions of BoLA-DRβ [30,31]. Through amino acid alignment from all BoLA-DRB3 types identified in this study, we found four (9, 47, 78, and 85) out of 17 positions with biallelic polymorphisms, and the remaining 13 positions accommodate more than two amino acid variants (Figure 1A). An association study between antigen recognition sites in peptide-binding pockets with BLV-induced lymphoma indicated that Q^9^, G^13^, L^26^, H^30^, F^47^, S^57^, R^70^, R^71^, E^74^, V^78^, and V^86^ were resistance amino acids (OR ranging from 0.201–0.554); in contrast, E^9^, T^11^, K^13^, Y^30^, Y^47^, D^57^,E^70^, K^71^, A^74^, Y^78^, and G^86^ were associated with lymphoma susceptibility (OR ranging from 1.832–3.665) (Figure 1B, only statistically significant amino acids are shown).

**Figure 2:**
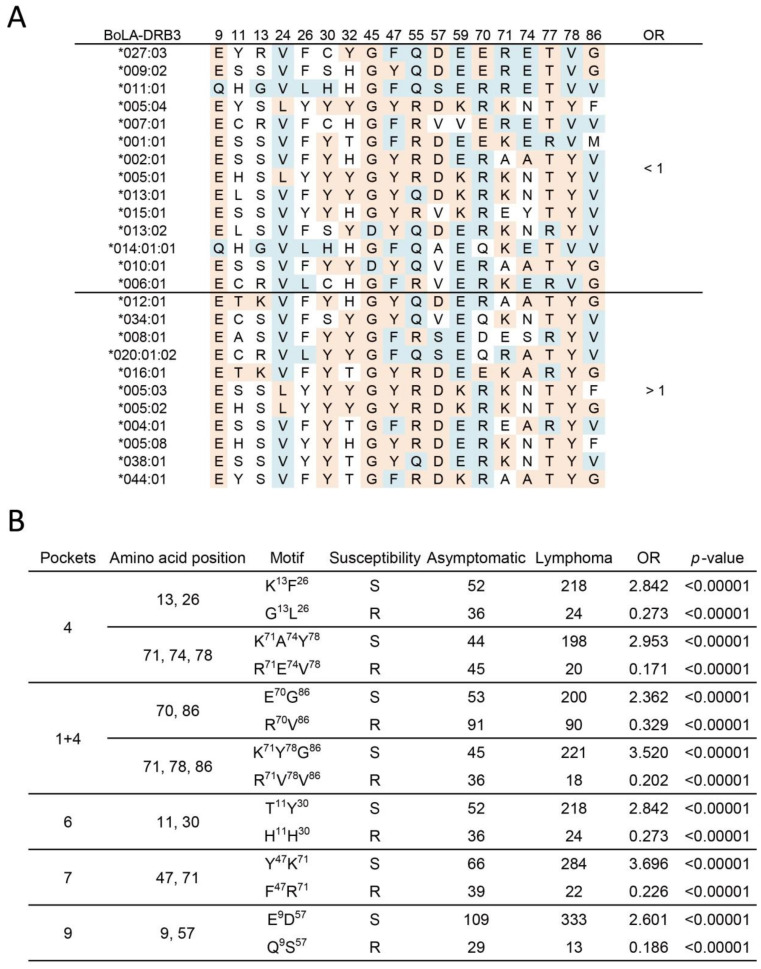
Association of amino acid motifs, which are constituted by antigen recognition sites of peptide-binding pockets in BoLA-DRβ, with lymphoma. (**A**) Conservation of lymphoma Resistance/Susceptibility amino acid distribution encoded by the Resistance/Susceptibility *BoLA-DRB3* allele. * indicates each allele. The order is ranked using Odds ratios (OR) determined in Table 1. *BoLA-DRB3* with OR < 1 are considered potential resistance alleles, whereas those with OR > 1 are considered susceptibility alleles. Resistance or susceptibility amino acids are indicated in blue and red, respectively. (**B**) Association of BLV-induced lymphoma with the combination of amino acid residues. Amino acid combinations and their localizations (pockets) are shown. Resistance amino acid combination: OR < 1; Susceptibility amino acid combination: OR > 1. The association study was performed using Fisher’s exact test.

The MHC bound peptide is determined by the properties of MHC-binding pockets, which are constituted by multiple antigen recognition sites. Therefore, it is important to note the association at the amino acid motif level. We further analyzed the effect of amino acid motifs, which included antigen recognition sites of the BoLA-DRβ chain. We observed that amino acid residues (Figure 1B), encoded by *BoLA-DRB3* in the potential resistance allele group (14 alleles, OR < 1, indicated in blue) or susceptibility allele group (11 alleles, OR > 1, indicated in red), were aligned (Figure 2A). Interestingly, the motifs associated with BLV-induced lymphoma were found in all BoLA-DRβ pockets-1, 4, 6, 7, and 9 (Figure 2B, only significant motifs are shown). In pocket 4, amino acid motifs 13, 26, 71, 74, and 78 were found to associate with lymphoma susceptibility. The motif K^13^F^26^ and K^71^A^74^Y^78^ were susceptible to lymphoma (OR = 2.84 and 2.95, respectively), whereas G^13^L^26^ and R^71^E^74^V^78^ represented resistance motifs (OR = 0.273 and 0.171, respectively). In addition, the combination of amino acid 86, found in pocket 1, with amino acid 70 or motif 71, 78 of pocket 4 was associated with lymphoma susceptibility (E^70^ G^86^, OR = 2.36; Y^71^G^78^ G^86^, OR = 3.52). In contrast, R^70^V^86^ and R^70^V^71^V^78^ represented the resistance amino acid combinations (OR = 0.329 and 0.202, respectively). In pocket 6, amino acid motifs 11 and 30 showed susceptibility to lymphoma. T^11^Y^30^ was found to be a susceptibility motif (OR = 2.842) and H^11^H^30^ represented a resistance motif (OR = 0.273). For pocket 7, the relationship was found in motifs 47 and 71. Y^47^K^71^ was identified as a susceptibility motif (OR = 3.696), whereas F^47^R^71^ was a resistance motif (OR = 0.226). In pocket 9, a motif at positions 9 and 57 was identified. E^9^D^57^ was found to be susceptible to lymphoma (OR = 2.601), whereas Q^9^S^57^ was resistant to lymphoma (OR = 0.186).

### 2.4. 3D Structure and Electrostatic Charge Analysis of BoLA-DRβ Binding Pocket

**Figure 3:**
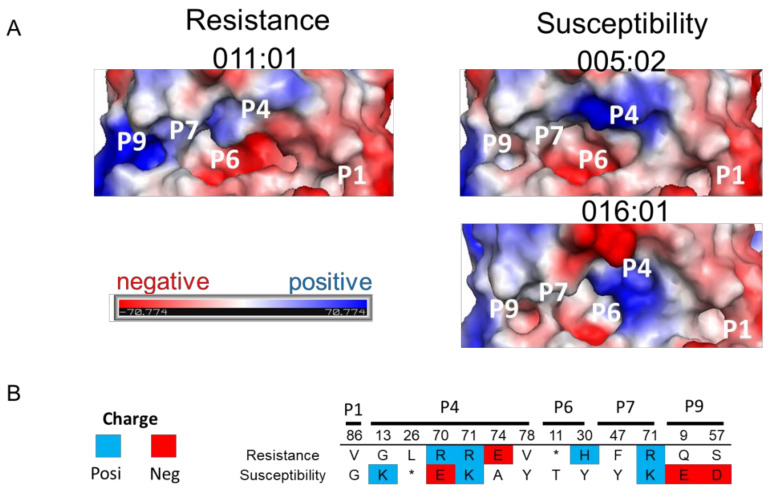
Electrostatic charge differences in binding pockets between lymphoma resistance and susceptibility BoLA-DRβ molecules. (**A**) Electrostatic potential of lymphoma resistance BoLA-DRβ molecule, DRB3*011:01, and susceptibility molecules, DRB3*005:02 and DRB3*016:01, is shown. Binding pockets 1, 4, 6, 7, and 9 are indicated. Negative charge is indicated in red, and positive charge is indicated in blue. (**B**) Lymphoma resistance/susceptibility amino acid charges. Positively charged amino acids are indicated with a blue background and negatively charged amino acids are indicated with a red background.

The electrostatic charge of the MHC binding pocket is reportedly associated with disease susceptibility [32,33]. The underlying mechanism is influenced by the interaction of the MHC binding pocket with specific bound peptides and the subsequent immunoreaction [34]. We investigated the charged potential of resistance and susceptibility BoLA-DRβ to determine whether electrostatic charged BoLA-DRβ binding pockets related to BLV-induced lymphoma (Figure 3A). Interestingly, a major charge difference was identified at binding pocket 9 between resistance and susceptibility BoLA-DRβ. A positive electrostatic charge was found in resistance type BoLA-DRβ, BoLA-DRB3*010:11 molecule. However, susceptibility BoLA-DRβ molecules, BoLA-DRB3*005:02 and BoLA-DRB3*016:01, were neutrally charged in binding pocket 9. These results are in line with the amino acid properties within binding pocket 9. Negatively charged amino acids, E^9^ and D^57^, were found in susceptibility BoLA-DRβ, potentially altering the positively charged environment, resulting in a neutral charged environment (Figure 3B).

## 3. Discussion

Herein, we report on the first association study between *BoLA-DRB3* (BoLA-DRβ) polymorphisms with BLV-induced lymphoma in Japanese black cattle at the DNA, amino acid, and electrostatically charged binding pocket levels. Here, DRB3*011:01 was identified as a resistance allele, whereas DRB3*005:02 and DRB3*016:01 were identified as susceptibility alleles. Interestingly, we demonstrated that antigen recognition sites of BoLA-DRβ at positions 9, 11, 13, 26, 30, 47, 57, 70, 71, 74, 78, and 86, as well as the amino acid motifs that covered all peptide-binding pockets, were related to lymphoma susceptibility. Electrostatic charge potential analysis found that pocket 9 of the resistance DRB3*011:01 allele encoding BoLA-DRβ was positively charged; in contrast, a neutral charge was observed in both DRB3*005:02 and DRB3*016:01. Our results not only identified *BoLA-DRB3* polymorphism susceptibility in Japanese black cattle but contribute to understanding the mechanisms underlying how *BoLA-DRB3* polymorphisms affect lymphoma susceptibility, thereby providing useful information for future vaccine development.

The association of *BoLA-DRB3* polymorphisms to BLV PVL varies in different cattle breeds or regions. Our findings show that, in Japanese black cattle, DRB3*011:01 represents a resistance allele with frequency 6.3%, whereas DRB3*005:02 and DRB3*016:01 represent susceptibility alleles with frequency 3.6% and 36.3%, respectively, among all allele populations in relation to BLV lymphoma. Previously, we found that DRB3*010:01 and DRB3*011:01 were lymphoma resistance alleles, but no susceptibility allele was found in Holstein cows in Japan [19]. In Iranian Holstein cows, BoLA-DRB3*018:02, DRB3*032:02, and DRB3*009:01 alleles are associated with susceptibility to BLV-induced lymphoma, whereas DRB3*001:01 and DRB3*011:01 are involved in lymphoma resistance [29]. DRB3*011:01 was identified as a resistance allele in all three independent studies; however, other resistance/susceptibility alleles are varied in different cattle breeds or regions. The discrepancies may be due to the allele distribution diversities in different breeds/regions of cattle. For example, the Holstein resistance allele, DRB3*001:01, also showed the resistance tendency in Japanese black cattle but did not reach a significant level, as only few cattle carried this allele in the Japanese cattle population. Furthermore, we did not find any Japanese black cattle harboring the Holstein susceptibility allele BoLA-DRB3*018:02, DRB3*032:02, and DRB3*009:01 in the current study. Therefore, association studies should address *BoLA-DRB3* polymorphisms and BLV-induced lymphoma in different regions or breeds of cattle.

The BoLA-DRβ molecule presents a peptide antigen for T cell recognition and initiates the immune response. There are five binding pockets of BoLA-DRβ, namely, pockets 1, 4, 6, 7, and 9, responsible for the interaction with the amino acids of antigen peptides at the corresponding positions [35]. These binding pockets shape the species of bound peptide as well as the susceptibility to specific diseases [34,35,36]. We previously found that amino acids 70 and 71 at pocket 4 of OLA DRB1 related with BLV-induced lymphoma [26]. Animals with the resistance motif at pocket 4 strongly expressed IFN-γ, which is known as a marker of Th1 response, suggesting the role of binding pocket polymorphisms in the determination of immune responsiveness and BLV-related disease susceptibility [27]. In the current study, in addition to amino acids 70 and 71 at pocket 4, we found that amino acids 9, 11, 13, 26, 30, 47, 57, 70, 71, 74, 78, and 86, which covered all binding pockets 1, 4, 6, 7, and 9, were associated with BLV-induced lymphoma susceptibility. Furthermore, through motif association study, we confirmed that two amino acid motifs: first, 13 and 26, and second, 71, 74, and 78, at pocket 4 were related with lymphoma susceptibility. The correlation was also identified in the amino acid combination of 70 and 86 and 71, 78, and 86 at binding pockets 1 and 4, respectively. At pocket 6, motif 11 and 30; pocket 7, motif 47 and 71; and pocket 9, motif 9 and 57 were found to be associated with BLV-induced lymphoma. As the susceptibility motifs were found in different binding pockets, it is likely that there is more than a single conserved type of BoLA-DRβ that could affect BLV-induced lymphoma susceptibility. Besides, the motif patterns which susceptible/resistant to lymphoma development we identified, could contribute to the prediction of susceptibility of some rare alleles, which could not be evaluated in allele association studies owing to their low frequencies. However, whether cattle with these specific motifs could bind with different peptides and induce differential immune responses still requires further investigation.

The electrostatic protein charge affects protein-protein interactions [37]. In the case of MHC, the charge of the MHC binding pocket affects peptide binding preference and therefore influences disease susceptibility. For example, positively charged binding pockets of the HLA-DQ2 molecule promote its ability to accommodate peptides with negatively charged anchor residues compared with other HLA-DQ molecules without positively charged binding pockets [28]. This is a key factor in allowing the HLA-DQ2 molecule to present gluten-derived peptides that are rich in prolines and glutamates [28]. Similarly, *HLA-DRB1* polymorphisms lead to differential electrostatic charges of binding pocket 9 and are thus related to the susceptibility to primary sclerosing cholangitis [38]. In this study, we found two lymphoma susceptibility molecules, DRB3*005:02 and DRB3*016:01, which were neutrally charged in binding pocket 9; whereas the resistance BoLA-DRβ molecule, DRB3*011:01, carries a positive charge. This electrostatic charge variation may allow the recognition of different peptide antigens and thus exert a differential immune reaction against BLV-induced lymphoma. Although we found that there were amino acid motifs associated with BLV-induced lymphoma in all binding pockets (1, 4, 6, 7, and 9), pocket 9 showed a major difference in electrostatic potential between resistance and susceptibility groups. This result implies that there might be other property differences in addition to that in electrostatic potential, such as in hydrophobicity of binding pockets, that affect BLV-induced lymphoma susceptibility. Interestingly, we found that the BoLA-DRB3*010:01 molecule, which is a lymphoma resistance type identified in Holstein cows, also carries a positive charge in pocket 9, in line with the current study (data not shown). However, BoLA-DRB3*010:01 was not categorized as a resistance allele in this study, suggesting the possibility of other host factors affecting the susceptibility to BLV-induced lymphoma. 

The development of BVL-induced lymphoma is a complex result caused by both viral and host factors in addition to *BoLA-DRB3*. For example, in the viral factors, BLV provirus integration close to cancer-driver sites or transcriptionally active regions influences host gene expression [39,40]. The viral accessory proteins, Tax and G4, are reported as causative agents for cell transformation [41,42]. For host factors, p53 mutation and the polymorphisms of tumor necrosis factor-α are related to lymphoma development [43,44,45]. Besides, the deregulation of lymphocyte homeostasis, which is characterized by the downregulation of cell turnover rate, is known to lead to leukemia [5,46]. Recently, we found that the expression levels of DNA mismatch repair genes *MSH2* and *EXO1* were associated with BLV-induced lymphoma, implying that the accumulation of DNA mutations is one of the mechanisms causing disease onset [47]. Furthermore, an arginine-N-methyltransferase, PRMT5, has shown positive correlation with BLV infection with a high pro-viral load and lymphoma stage. Downregulation of PRMT5 expression impaired BLV gene expression and pathogenicity [48]. The factors mentioned above potentially working together with *BoLA-DRB3* polymorphisms contribute to BLV-induced lymphoma development. 

Taken together, in addition to the BLV-induced lymphoma association study regarding *BoLA-DRB3* alleles, amino acid and structure level analyses augment the understanding of how BoLA-DRB3 polymorphisms affect lymphoma susceptibility. These results are not only helpful for cattle breeding selection but for future vaccine development against BLV-induced lymphoma.

## 4. Materials and Methods

### 4.1. Sample Collection and Diagnosis

Blood samples from 106 BLV-infected but clinically normal purebred Japanese black cattle (asymptomatic cattle) and 227 BLV-infected purebred Japanese black cattle with lymphoma (lymphoma cattle) which were selected from a nationwide survey (12 out of 48 prefectures) across Japan were used in this study, and the genomic DNA and plasma from the peripheral blood were isolated. Asymptomatic cattle samples were collected from farms and the infection was confirmed by anti-BLV gp51 ELIAS. Lymphoma cattle samples were collected from slaughterhouses and the infection were confirmed by anti-BLV gp51 ELIAS and some of the sample tested together with southern blotting and PCR of BLV pro-viral genomes. As lymphoma onset in cattle occurs, on average, seven years after BLV infection [49], all asymptomatic cattle were > 9 years old to reduce the likelihood of collecting potential lymphoma cattle. The age difference between lymphoma and asymptomatic cattle was therefore compensated for further association study. Asymptomatic cattle sample were collected from farms and the subclinical stage of BLV infection was diagnosed according to the lymphocyte count (cells/μL) and the age of each cow (≤5500 = normal, between 5500 to 7500 = suspected lymphocytosis and ≥7500 = lymphocytosis). Asymptomatic cattle were defined as BLV-infected but clinically and hematologically normal; PL cattle were defined as BLV-infected but clinically normal cattle with an increase in the number of apparently normal B lymphocytes. In this study, only samples from asymptomatic cattle were used for further analysis. This study was approved by the Animal Ethical Committee, and the Animal Care and Use RIKEN Animal Experiments Committee (approval number H29-2-104). Cattle lymphoma status was diagnosed using both gross observation of neoplastic tissues in lymph nodes and histological observation in heart, lung, liver, kidney, spleen, intestines and lymph node in the body. In addition, atypical mononuclear cells in blood sample together with genomic southern blotting for testing disease progression were used for confirmation of some cattle samples [50]. 

### 4.2. BLV Infection Determination Using Enzyme-Linked Immunosorbent Assays (ELISA)

The anti-BLV gp51 antibody was measured using an anti-BLV antibody ELISA Kit (JNC, Tokyo, Japan), according to the manufacturer’s instructions.

### 4.3. BoLA-DRB3 Genotyping

*BoLA-DRB3* alleles were determined using the PCR-sequence-based typing (SBT) method, as previously described [51]. Briefly, *BoLA-DRB3* exon 2 was amplified via single-step PCR using the *DRB3* forward (5′-CGCTCCTGTGAYCAGATCTATCC-3′) and reverse (5′-CACCCCCGCGCTCACC-3′) primer set. The PCR products were purified using the ExoSAP-IT PCR product purification kit (USB Corp., Cleveland, OH, USA) and then sequenced using the ABI PRISM BigDye1.1 Terminator Cycle Sequencing Ready Reaction Kit (Applied Biosystems, Foster City, CA, USA). The sequence data were then analyzed using Assign 400ATF ver. 1.0.2.41 software (Gonexio Genomics, Fremantle, Australia) to determine the *BoLA-DRB3* genotype. 

### 4.4. Characterization of Amino Acid Properties

Amino acid charge property of the BoLA-DRβ binding pocket was characterized according to international ImMunoGeneTics information system [52].

### 4.5. 3-Dimensional (3D) Protein Structure Modeling of BoLA-DRB3 Molecules

All *BoLA-DRB3* sequences were downloaded from the IPD-MHC database (https://www.ebi.ac.uk/ipd/mhc/group/BoLA/) (accessed on 4 April 2021). The amino acid sequence alignment was performed using Mega X software [53]. Graphical 3D structures and electrostatic surface potential of BoLA-DRB3 molecules were determined with PyMOL 2.4 (Schrodinger LLC, New York, NY, USA). The structural models of all *BoLA-DRB3* molecules were constructed based on the crystal structure of HLA-DRB1: Protein Data Bank (PDB) ID: 1dlh [54].

### 4.6. Association Study and Statistical Analysis

An association study based on Fisher’s exact test was performed by comparing the allele, genotype, or amino acid frequencies between asymptomatic and lymphoma cows. The results were penalized with the Bonferroni correction procedure to correct for false positive rate. The alleles or genotypes with odds ratios (OR) < 1 were categorized as resistance alleles or genotypes. In contrast, those with OR > 1 were defined as susceptibility alleles or genotypes. All calculations were performed using Prism 6 (GraphPad, San Diego, CA, USA).

## 5. Conclusions

This is the first report demonstrating the association between *BoLA-DRB3* polymorphisms and BLV-induced lymphoma in Japanese black cattle. Through comprehensive analysis of the association at DNA, amino acid, and structure levels, the following three main results were obtained in the present study. First, BoLA-DRB3*011:01 was identified as a lymphoma resistance allele, whereas BoLA-DRB3*005:02 and BoLA-DRB3*016:01 were identified as susceptibility alleles. Second, an amino acid association study indicates that Q^9^, G^13^, L^26^, H^30^, F^47^, S^57^, R^70^, R^71^, E^74^, V^78^, and V^86^ are resistance amino acids; in contrast, E^9^, T^11^, K^13^, Y^30^, Y^47^, D^57^, E^70^, K^71^, A^74^, Y^78^, and G^86^ are associated with lymphoma susceptibility. Third, the structure and electrostatic potential modeling shows that the binding pocket 9 of the resistance BoLA-DRB3 molecule is positively charged. Conversely, it is neutrally charged in susceptibility BoLA-DRB3 molecules. Further studies are warranted to determine whether resistance and susceptibility *BoLA-DRB3* molecules can bind with different peptide antigens and trigger differential immune responses against BLV and related pathogenesis.

## Figures and Tables

**Table 1:** Association of the *BoLA-DRB3* allele with Bovine leukemia virus (BLV)-induced lymphoma.

Allele	Asymptomatic (n. = 212) ^1^	Lymphoma (n. = 454)	OR ^2^	95% CI ^3^	*p*-Value	Susceptibility ^4^
*001:01	9	8	0.405	0.154–1.064	0.0677	-
*002:01	18	17	0.419	0.212–0.831	0.0147	(R) ^5^
*004:01	0	1	1.406	0.057–34.653	1	-
*005:01	1	1	0.466	0.029–7.483	0.5356	-
*005:02	1	23	11.260	1.510–83.945	0.0014	S
*005:03	3	31	5.106	1.543–16.894	0.0021	(S)
*005:04	2	1	0.232	0.021–2.571	0.2391	-
*005:08	0	3	3.295	0.169–64.072	0.5552	-
*006:01	1	2	0.934	0.084–10.354	1	-
*007:01	7	5	0.326	0.102–1.040	0.0606	-
*008:01	1	4	1.876	0.208–16.884	1	-
*009:02	7	2	0.130	0.027–0.629	0.0059	(R)
*010:01	27	45	0.754	0.454–1.253	0.2855	-
*011:01	29	13	0.186	0.095–0.366	<0.00001	R
*012:01	8	20	1.175	0.509–2.713	0.8369	-
*013:01	1	1	0.466	0.029–7.483	0.5356	-
*013:02	13	20	0.705	0.344–1.447	0.3426	-
*014:01:01	7	11	0.727	0.278–1.903	0.6086	-
*015:01	29	34	0.511	0.302–0.864	0.0024	(R)
*016:01	44	198	2.953	2.019–4.319	<0.00001	S
*027:03	2	0	0.093	0.004–1.938	0.1010	-
*020:01:02	1	4	1.876	0.208–16.884	1	-
*034:01	1	3	1.404	0.145–13.573	1	-
*038:01	0	3	3.295	0.169–64.072	0.5552	-
*044:01	0	4	4.245	0.228–79.214	0.3126	-

* indicates the allele; ^1^ n.: total allele number (= cattle number multiplied by 2); ^2^ OR: Odds ratio; ^3^ 95% CI: 95% confidence intervals; ^4^ Susceptibility: R = resistance; S = susceptibility after Bonferroni correction; ^5^ ( ): significant only before Bonferroni correction.

**Table 2:** Association of *BoLA-DRB3* genotype with BLV-induced lymphoma.

Genotype ^1^	Asymptomatic (n. = 106) ^2^	Lymphoma (n. = 227)	OR ^3^	95% CI ^4^	*p*-Value	Susceptibility ^5^
*001:01/*016:01	2	3	0.696	0.115–4.231	0.6551	-
*002:01/*005:03	1	3	1.406	0.145–13.681	1	-
*002:01/*010:01	5	2	0.180	0.034–0.941	0.0356	(R) ^6^
*002:01/*011:01	5	0	0.041	0.002–0.741	0.0031	(R)
*002:01/*016:01	1	7	3.341	0.406–27.507	0.4438	-
*005:02/*016:01	0	13	13.405	0.789–227.680	0.0115	(S)
*005:03/*012:01	0	4	4.289	0.229–80.387	0.311	-
*005:03/*016:01	0	10	10.283	0.597–177.152	0.0341	(S)
*010:01/*010:01	2	3	0.696	0.117–4.312	0.6551	-
*010:01/*011:01	3	3	0.460	0.091–2.317	0.3873	-
*010:01/*015:01	3	4	0.616	0.135–2.802	0.6837	-
*010:01/*016:01	7	19	1.292	0.526–3.175	0.6654	-
*011:01/*015:01	4	0	0.050	0.003–0.939	0.0099	(R)
*011:01/*016:01	7	4	0.254	0.073–0.886	0.0415	(R)
*012:01/*015:01	2	3	0.696	0.115–4.231	0.6551	-
*012:01/*016:01	0	5	5.265	0.289–96.103	0.1822	-
*013:02/*016:01	3	10	1.582	0.426–5.872	0.7621	-
*015:01/*016:01	10	15	0.679	0.295–1.567	0.3769	-
*016:01/*016:01	4	49	7.020	2.462–20.017	<0.00001	S
*016:01/*020:01:02	1	3	1.406	0.145–13.681	1	-

* indicates the genotype; ^1^ Only genotypes with frequency >1 are shown; ^2^ n.: total genotype number; ^3^ OR, Odds ratio; ^4^ 95% CI: 95% confidence intervals; ^5^ Susceptibility, R = resistance, S = susceptibility after Bonferroni correction; ^6^ ( ), significant only before Bonferroni correction.

## Data Availability

The data presented in this study are available on request from the corresponding author.
